# Models of Postural Control: Shared Variance in Joint and COM Motions

**DOI:** 10.1371/journal.pone.0126379

**Published:** 2015-05-14

**Authors:** Melissa C. Kilby, Peter C. M. Molenaar, Karl M. Newell

**Affiliations:** 1 Department of Kinesiology, The Pennsylvania State University, 23 Recreation Building, University Park, Pennsylvania, 16802, United States of America; 2 Department of Human Development and Family Studies, The Pennsylvania State University, 315 Health and Human Development - East, University Park, Pennsylvania, 16802, United States of America; 3 Department of Kinesiology, The University of Georgia, Athens, Georgia, 30602, United States of America; Purdue University, UNITED STATES

## Abstract

This paper investigated the organization of the postural control system in human upright stance. To this aim the shared variance between joint *and* 3D total body center of mass (COM) motions was analyzed using multivariate canonical correlation analysis (CCA). The CCA was performed as a function of established models of postural control that varied in their joint degrees of freedom (DOF), namely, an inverted pendulum ankle model (2DOF), ankle-hip model (4DOF), ankle-knee-hip model (5DOF), and ankle-knee-hip-neck model (7DOF). Healthy young adults performed various postural tasks (two-leg and one-leg quiet stances, voluntary AP and ML sway) on a foam and rigid surface of support. Based on CCA model selection procedures, the amount of shared variance between joint and 3D COM motions and the cross-loading patterns we provide direct evidence of the contribution of multi-DOF postural control mechanisms to human balance. The direct model fitting of CCA showed that incrementing the DOFs in the model through to 7DOF was associated with progressively enhanced shared variance with COM motion. In the 7DOF model, the first canonical function revealed more active involvement of all joints during more challenging one leg stances and dynamic posture tasks. Furthermore, the shared variance was enhanced during the dynamic posture conditions, consistent with a reduction of dimension. This set of outcomes shows directly the degeneracy of multivariate joint regulation in postural control that is influenced by stance and surface of support conditions.

## Introduction

The human muscular-skeletal system consists of multiple components at different levels that need to be coordinated in the service of action [1]. For example, in order to stand upright, torques at the various body joints must be applied and multi-joint actions coordinated in such a way that the total body’s center of mass (COM) position is stabilized against gravity [2]. However, a longstanding assumption has been that the whole body is swaying about the ankle joint with the remaining joints locked. Based on this assumption postural control has been modeled as a single link, inverted pendulum, whereas the center-of-pressure (COP = location of vertical ground reaction force) can be regarded as the control variable and the COM as the controlled variable [2, 3]. This simple mechanistic relationship has been supported by evidence that the difference between COP and COM is proportional to COM acceleration [2, 4].

The single inverted pendulum model has long been considered the fundamental and simplest model of postural control [2]. This assumption has led to the formulation of an *ankle strategy* as the primary source of control during human quiet stance [5–9]. However, studies of postural responses on a moving platform [5, 10, 11] have revealed that a hip strategy is also used in conjunction to the ankle strategy. Indeed, even without platform perturbation significant hip motion has been reported [4, 12–16] and a substantial role of the knee joint in quiet standing has also been revealed [17–20]. Additional experimental evidence against the single joint (ankle strategy) inverted pendulum model has been provided using the uncontrolled manifold (UCM) data analysis approach [20–23]. In general, the findings show that postural control is multivariate in nature, involving the many joint space degrees of freedom, leaving the inverted pendulum model as too simplistic to accommodate the control problem.

Therefore, in light of the multi-segmented body and the fact that the total body’s COM is the weighted average of segmental center-of-mass positions, mechanical multi-link models of postural control as opposed to the single link, inverted pendulum [2, 6, 19, 20] have been derived to gain deeper insight into the nature of balance control processes during upright stance. A central focus in this line of research has been to address the relation between the functional degrees of freedom (DOF) and the joint mechanical DOF of the postural control system. Existing inferences about the functional joint DOF are based on the contribution of body joint motions to the maintenance of upright stance. The contribution of each joint to postural control has largely been assessed *indirectly* by the amount of COP motion [2], the joint motion variability [4, 15, 22], the magnitude of net joint torques [24], and the bivariate correlation between each of the joint angular displacements and the COM displacement [4, 14].

The listing of methods emphasizes the role of variances and covariance to quantify postural motion. In addition, the strength of correlation between each joint and the COM has been extensively used to determine the importance of each joint motion during upright stance [4, 14]. Gatev and colleagues [14] showed that only the ankle joint was highly correlated with the motion of the COM in the sagittal plane, whereas the knee and hip joints were not. Gage et al. [4] found that the leg segment angle correlated more highly with the COM than the ankle joint alone. It was concluded that compensatory knee movement plays a significant role in quiet stance.

Federolf et al. [25] used a principal component analysis (PCA) to decompose the nature of the multivariate input to postural control. They showed that during bipedal quiet stance the first principal component was generally dominated by the ankle sway in the sagittal plane. More challenging postures like tandem or single leg stance showed highly individual postural strategies and the number of principal components that accounted for most of the total variance increased [26, 27]. Thus, with increasingly challenging task constraints postural strategies become more complex and multi-DOF are involved in more active roles [10, 26, 28, 29].

Several studies have built upon the extant posture models and characterized the coordination patterns among the principal joint motions, especially between the ankle and hip joints [2, 5, 30–33]. Creath and colleagues [16] performed a coherence and co-phase analysis and found anti-phase coupling of ankle and hip above 1Hz and in-phase coupling below 1 Hz. Aramaki et al. [15] found an inverse relationship between the angular accelerations of the ankle and hip in order to minimize COM acceleration. Our previous work [28] showed that the COP-COM coherence in low-frequency ranges was larger and more consistent across various stance conditions than the coupling between the different joints (all possible combinations of ankle, knee, hip and neck). Therefore, following a dynamical system view, it was suggested that individual joint couplings of a multi-linkage posture model are embedded within the higher-order collective variable of COP-COM coupling.

This paper reports an experiment that was set up to examine the relation between joint motion and the motion of COM through a canonical correlation analysis (CCA) [34–36]. This is a general approach that can reveal the linear structure between COM sway in three-dimensional space and joint motions. CCA is based on simultaneous singular value (eigenvalue) decomposition of two multivariate data sets in such a way that the component scores associated with the first eigenvector of the first data set has maximum correlation with the component scores associated with the first eigenvector of the second data set. Given the first eigenvectors, the component scores associated with the second eigenvectors (which are orthogonal to the first eigenvectors) again have maximum correlation, etc.

In this study CCA was used to decompose the total variance of the data into functions of decreasing order that capture the shared variance of the motion of individual and combinations of joint components *with* the variance of the 3D-COM as a function of different model assumptions regarding joint inputs. Through this approach we examined *directly* in what way multi-joint DOF posture models represent postural control strategies. We examine what statistically is labeled as the redundancy index to give a global measure of the amount of variance in each linear combination that can be explained by the two sets. Note the use of the term redundancy index in CCA is different from the meaning embedded in the motor control literature through Bernstein (1967). In our context, the CCA redundancy index quantifies the shared variance of the motions of the joint components with that of the 3D COM. We also report the cross-loadings of each variable in both sets of variables to determine the principal COM sway direction and the contribution of each joint to the optimal linear structure between the sets.

More specifically, we compared posture models that were based on the different mechanical DOF models of postural control [4, 5, 19, 20, 22], namely, ankle-model (2 DOF), ankle-hip-model (4 DOF), ankle-knee-hip-model (5 DOF) and ankle-knee-hip-neck model (7 DOF). Except for the knee joint each joint motion was given 2 DOF (anterior-posterior (AP) and medial-lateral (ML) joint motions). In addition, we used CCA model selection approaches to statistically derive the optimal model [37, 38] as opposed to the theoretically motivated posture models. Inferences about the *true* functional DOF of the postural control system will be based on the CCA model selection outcomes, the amount of shared variance between joint and 3D COM motions and the cross-loading patterns. Furthermore, previous work has shown that control mechanisms during bipedal quiet stance differ from perturbed stance or challenged stances as, for example, in standing on one leg [5, 25, 29]. Therefore, we also compared the different posture models in quiet bipedal stance as well as in more challenging postures (standing on one leg and/or on a foam surface) in order to determine the direct fit of the different multi-DOF posture models to the control of upright stance and motion of the COM [28].

In summary, this study investigated how the multiple joint space DOFs are organized in different upright stances of postural control. To this aim established posture models with different mechanical DOF are compared with each other in terms of their shared variance with the motion of COM using canonical correlation analysis [34–36]. On this direct basis, we determined the relative contribution of joint motions to the maintenance of upright stance and the principal direction of COM sway [4, 19, 20]. These features were examined under increasingly complex posture tasks (bipedal stance, one-leg stance, voluntary AP and ML sway), including standing on a compliant foam surface [16, 29, 39] and the standard rigid ground support surface.

## Methods

### Participants

Twelve healthy participants (28.6 ± 3.5 years, 6 females and 6 males) were recruited for this study. The experimental protocol was approved by the Institutional Review Board of the Pennsylvania State University. After giving written informed consent, participants started with the experimental procedures.

### Apparatus

We used seven infrared cameras and the Qualisys Track Manager Software (Qualisys AB, Gothenburg, Sweden) to record the 3D motion of 20 passive reflective markers at a sample rate of 100Hz. Ground reaction force data were also collected at 100Hz using two adjacent AMTI (American Mechanical Technology, Inc., Watertown, MA) force platforms. The two systems were temporally synchronized. In addition, we used two medium firm polyurethane foam pads of 10 cm height (same length and width as the force platforms).

### Tasks and procedures

The 20 reflective markers were attached to the following landmarks of the respective body segment: 3^rd^ metatarsal, heel, lateral malleolus, lateral femoral epicondyle, greater trochanter, iliac crest, acromion process, lateral humeral epicondyle, dorsal wrist (between radial and ulnar styloid), and the lateral aspect of the head (anterior to ear canal).

The participants completed 3 trials that lasted for 35 s in each of 4 different stances (two-leg, one-leg, voluntary AP and ML sway) on both a firm and more compliant (foam) surface, totaling 8 experimental conditions. The order of foam and no foam blocks was randomized across participants. In addition, the order of stance conditions within each block was randomized. During two-leg stance and AP and ML sway conditions participants stood in an upright posture with the feet hip width apart, each foot placed on one of two force platforms. We marked the foot position to avoid variation across trials and conditions. The instruction for one-leg and two-leg stances was to stand as still as possible. For one-leg stance participants were asked to stand on their preferred supporting leg. For AP and ML sway participants were asked to voluntarily sway at the sound of a 0.45 Hz metronome. The participants were free to choose their preferred sway amplitude. The task goal of AP sway was to naturally sway back and forth. The instruction for ML sway was to naturally shift weight from one leg to the other. During all conditions participants were standing barefoot with their arms crossed above their chest. Participants were asked to look at a focal point positioned at eye level 3m in front of the platforms.

### Data analysis

Data were analyzed in Matlab (MathWorks, Natick, MA). The total body COM position was calculated as the weighted sum of the center of mass positions of the head, upper arms, forearms/hands, thorax/abdomen, pelvis, thighs, shanks and feet. In addition, the net COP (COPnet) of the two force platforms was calculated from the ground reaction force data. The mean velocities of the 2D COPnet and 2D COM (AP and ML directions) paths were calculated as traditional postural stability indices [40].

**Fig 1:**
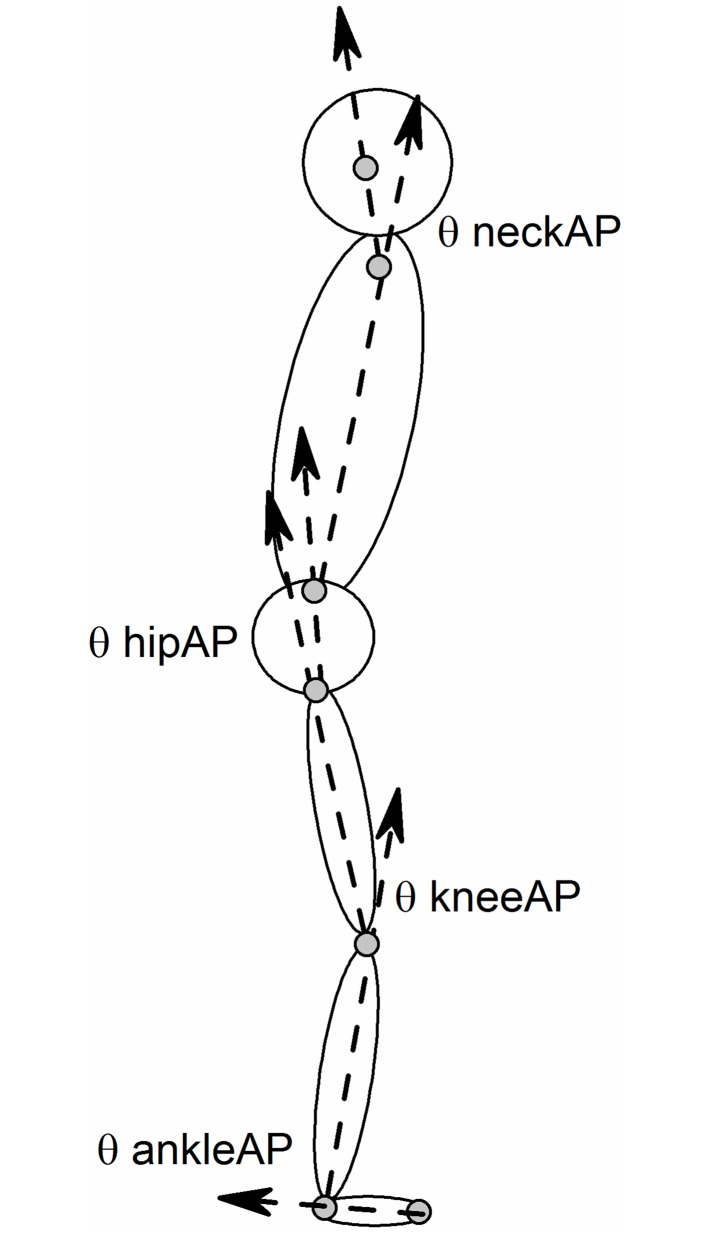
Schematic illustration of the CCA input joint angles in anterior-posterior (AP) direction (ankleAP, kneeAP, hipAP and neckAP).

Based on the markers positioned at the endpoints of the body segments we defined vectors of the foot, shank, thigh, pelvis, thorax/abdomen and head, similar to Hsu and colleagues [20]. Subsequently, the following joint angles in the sagittal plane: ankleAP, kneeAP, hipAP and neckAP (Fig 1) and in the frontal plane: ankleML, hipML and neckML were computed. The planar angles were computed using the general trigonometric relationship of the tangent:

$$\theta =\tan^{-1}\frac{\left|{\vec{v}}_{1}\times {\vec{v}}_{2}\right|}{{\vec{v}}_{1}\cdot {\vec{v}}_{2}}$$ (1)

where ${\vec{v}}_{1}$ and ${\vec{v}}_{2}$ are the 3D vectors of two adjacent body segments. Given the one-leg stance condition, ankle, knee and hip joint angles were only computed for the preferred supporting leg. Circular statistics was used to report the circular SD of the joint angular motions as a descriptive statistic of the joint motion variability [41].

**Fig 2:**
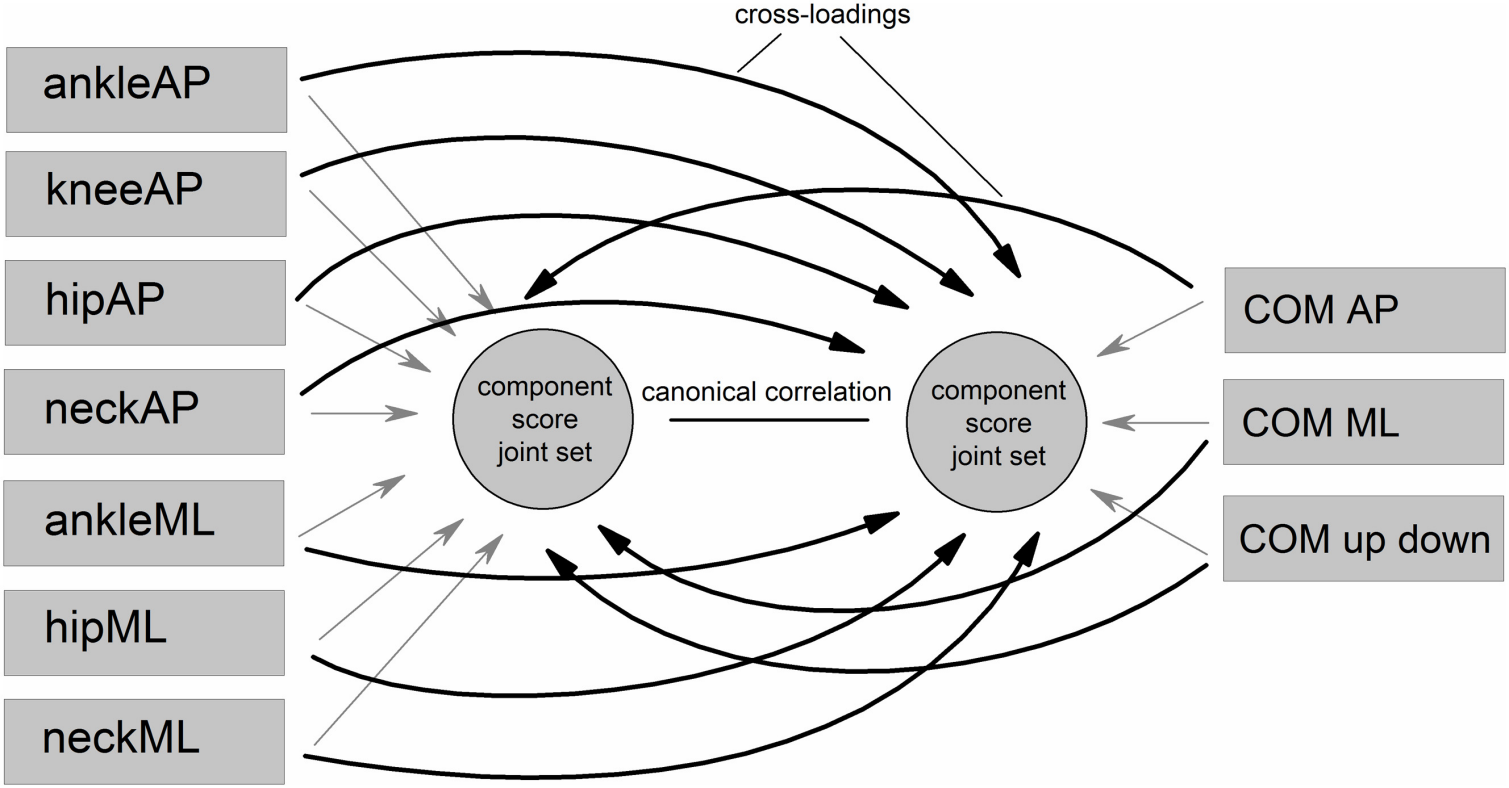
Basic procedures of the canonical correlation analysis (CCA) in conceptual diagram form.

**Fig 3:**
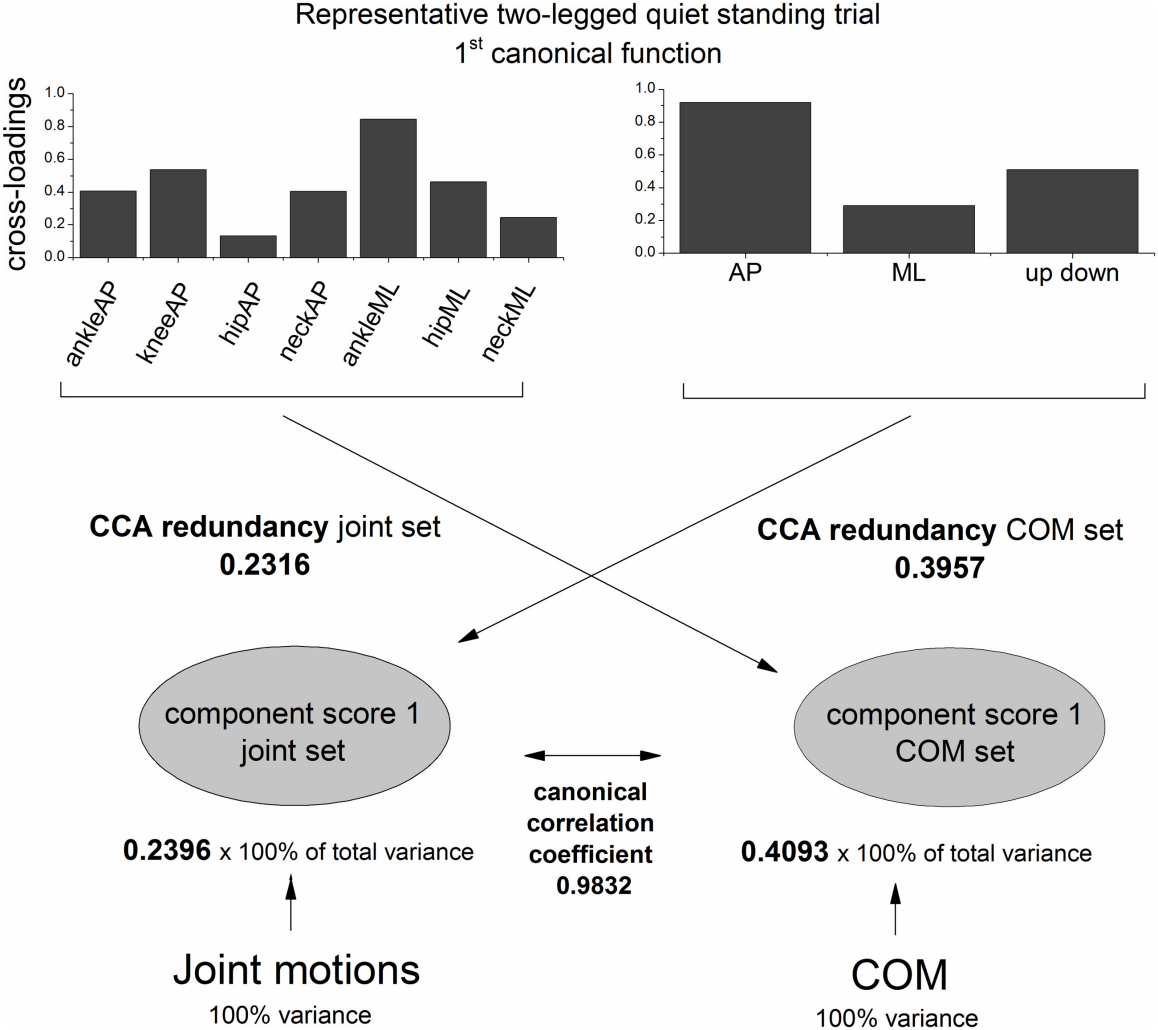
Cross-loadings, CCA redundancy, canonical correlation coefficient and amount of variance in each component score of the first canonical function of one representative trial during two-legged quiet stance.

We used a canonical correlation analysis (CCA) [34–37] to interrelate multiple joint angles (joint set = set 1) to the 3D COM position (COM set = set 2). Figs 2 and 3 illustrate the basic procedures of the canonical correlation analysis in conceptual diagram form.

Set 1 with *p* variables and *n* observations is represented by a *n*×*p* random variable *X*. Set 2 with *q* variables and *n* observations is represented by a *n*×*q* random variable *Y*. CCA creates *d* = min(*rank*(*X*), *rank*(*Y*)) pairs of *n*×1 linear combinations (= component scores) *U* and *V* of the original variables from each set:

$$U_{i}=Xa_{i}\text{ and }V_{i}=Yb_{i}$$ (2)

where *i* = 1,…, *d*, *a*_*i*_ and *b*_*i*_ are *p*×1 and *q*×1 coefficient vectors. Let *S* be the total (*p* + *q*, *p* + *q*)-dimensional variance-covariance matrix of *X* (set 1) and *Y* (set 2):

$$S=\left[\begin{array}{cc} S_{11} & S_{12}\\ S_{21} & S_{22} \end{array}\right]$$ (3)

Using singular value decomposition the eigenvalues in decreasing order and the corresponding eigenvectors of

$$A_{p}=S_{11}^{-1}S_{12}S_{22}^{-1}S_{21}\text{ and }A_{q}=S_{22}^{-1}S_{21}S_{11}^{-1}S_{12}$$ (4)

are obtained. The *i*th eigenvector of *A*_*p*_ constitutes the *a*_*i*_ coefficients and the *i*th eigenvector of *A*_*q*_ the *b*_*i*_ coefficients. The canonical correlations are derived from the first *d* eigenvalues *λ*_i_. The canonical correlation *r*_*i*_ is the square root of *λ*_*i*_. The eigenvalues of *A*_*p*_ and *A*_*q*_ are the same and either one can be used to obtain the canonical correlation.

$$r_{i}=\sqrt{\lambda_{i}}$$ (5)

CCA was performed using standardized data, therefore *S* can be replaced by the correlation matrix *ρ*. The significance of each canonical function (pairs of *U* and *V*) was assessed using F-statistics.

Fig 3 highlights that only a proportion of total variance of the data is represented by the component score associated with the first eigenvector of the respective set. The value represents an average proportion of total variance of the original variables. The CCA redundancy index of each set (CCA redundancy COM set and CCA redundancy joint set) can be obtained by multiplying the average proportion of total variance by the squared canonical correlation coefficient. It quantifies the amount of variance represented by the component score associated with the *i*th eigenvector of set 1 that can be explained by the component score associated with the *i*th eigenvector of set 2 and vice versa. Similar to *R* ^2^ in multiple regression it is the *shared variance* between the two sets, that is, how much variation in the COM position can be predicted by variation in joint angles. In this study we report the sum of the CCA redundancy values of the first two component score pairs as they are assumed to capture the most important variance. This index was labelled *total CCA redundancy*. A pair of component scores associated with the *i*th eigenvectors of the two sets is commonly termed the *i*th canonical function [34].

Furthermore, we computed the cross-loading of each variable in both sets. The cross-loadings are the bivariate correlations between each original variable and the component score of the other set. Here, a high squared cross-loading generally indicates that a change in angular motion was matched by a change in COM position. However, there are no general guidelines for distinguishing high versus low cross-loadings [37]. Therefore, the interpretation of the cross-loadings is kept at a qualitative level. Note that the CCA redundancy index can also be obtained by averaging the squared cross-loadings. Fig 3 shows the squared cross-loadings and CCA redundancy of the first canonical function of one representative trial during two-legged quiet stance.

In this study model or variable selection was motivated both theoretically [2, 4, 5, 16, 19, 20, 22] and statistically [37, 38]. Based on existing literature three different subsets of joint angles of the full 7DOF-model were examined while the COM set was held constant (COMAP, COMML and COMupdown). The 7DOF-model contains all variables, that is, ankleAP, kneeAP, hipAP, neckAP, ankleML, hipML and neckML. The 2DOF-model (subset 1) includes ankleAP and ankleML joint angles, the 4DOF-model (subset 2) ankleAP, hipAP, ankleML and hipML, and the 5DOF-model (subset 3) ankleAP, kneeAP, hipAP, ankleML, and hipML.

On the other hand, similar to variable selection in regression analysis a simple sequential approach was chosen for statistical model building in CCA [37]. All variables of the 7DOF-model (7 variables in set 1 and 3 variables in set 2) were subject to this sequential method in order to test whether the full (10 variables) or a reduced model is the best model. The first step is to choose two variables (one from each set) from all possible *p*×*q* combinations that minimize Wilks’ lambda Ʌ:

$$\Lambda =\overset{d}{\underset{i=1}{\Pi }}(1-\lambda_{i})$$ (6)

The procedure only continues if this best combination is significant. The next step is to determine the variable of the remaining variables that minimizes partial lambda Ʌ_*partial*_:

$$\Lambda_{partial}=\frac{\Lambda_{full}}{\Lambda_{red}}$$ (7)

where lambda full Ʌ_*full*_ is based on the first two variables plus the potential new variable and lambda reduced Ʌ_*red*_ on the first two variables. The variable that minimizes Ʌ_*partial*_ enters the model if the following F-statistic that follows an F-distribution (*α* = 0.01 and *w*, *n* − p* − q*degrees of freedom) is significant:

$$F=\frac{\left(1-\Lambda_{partial}\right)}{\Lambda_{partial}}\cdot \left[\frac{\left(n_{}-p^{*}-q^{*}\right)}{w_{}}\right]$$ (8)

Where p* is the number of current variables in set 1 and q* in set 2. *w* equals p* if a *X* variable is tested and q* if a *Y* variable is tested. Ʌ_*red*_ and Ʌ_*full*_ are constantly being updated until either all possible variables are included in the model or the best potential new variable does not significantly improve the model fit.

Furthermore, a second variable selection method based on the total CCA redundancy of *X*, that is, the sum of the redundancy values of the first two canonical functions was applied [38]. The approach seeks to find the subsets X* and Y* that are smaller than the original sets and best represent the original shared variance between the two sets. As a first step the total CCA redundancy of *X* using the two original sets is computed as a reference value (*TRed*_*X*,*Y*_). Now the best subset Y* is sought. The best subset Y* (here containing 2 variables) of all possible variable combinations is the one that is closest to *TRed*_*X*,*Y*_ and, therefore, satisfies the following condition:

$$\min(TRed_{X,Y^{}}-TRed_{X,Y^{*}})$$ (9)

where *TRed*_*X*,Y*_ is the total CCA redundancy of *X* given Y*. Subsequently, the total CCA redundancy reference value for finding the best subset X* of *X* is updated to be *TRed*_*X*,Y*_. All possible variable combinations forming subsets X* (here containing 2 to 6 variables at a time) are tested and the one that satisfies:

$$\min\left[(TRed_{X,Y^{*}}-TRed_{X^{*},Y^{*}})\cdot \frac{p^{*}}{p}\right]$$ (10)

represents the best subset X*. Multiplication by $\frac{p^{*}}{p}$ normalizes the total CCA redundancy of X* to the full set *X*. Note that this normalization was also applied to report the CCA redundancy of the theoretically motivated 2DOF, 4DOF and 5DOF-models. Each analysis was performed on an individual trial basis.

### Statistics

To analyze the statistical effects of the traditional postural stability indices and the redundancy indices of the 7DOF-model we performed a two-way repeated measures ANOVA. The two factors were postural stance (4 levels) and foam (2 levels). For post hoc pairwise multiple comparisons we used the Bonferroni correction. Statistical analysis was performed in RStudio (The R Project for Statistical Computing).

## Results

### Variability of joint and COPnet/COM motion

**Fig 4:**
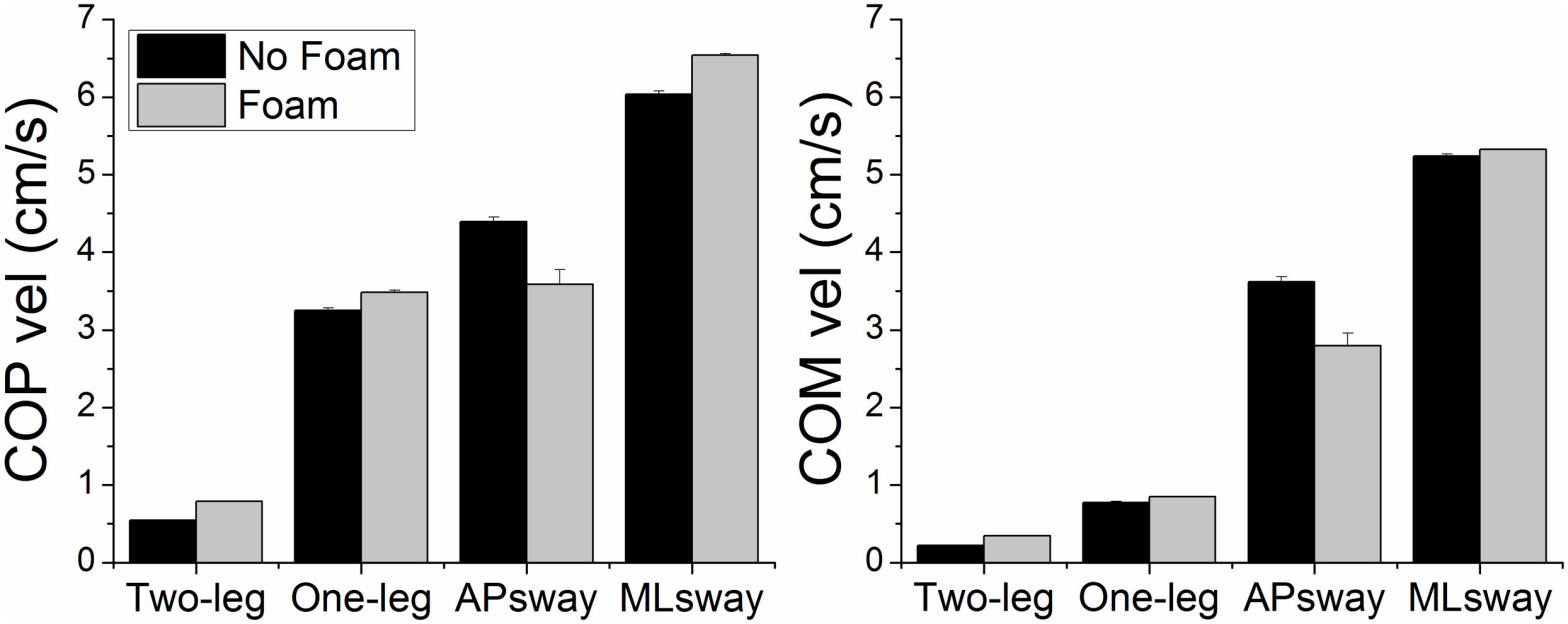
COPnet and COM velocities (group means ± SE) as a function of postural stance and surface of support condition.

Fig 4 shows the mean velocities of the COPnet and COM paths as a function of stance and surface of support condition. There was a significant main effect of postural stance for COPnet velocity (F_3,33_ = 130.75, p < 0.01) and for COM velocity (F_3,33_ = 213.62, p < 0.01). All pairwise comparisons were significant. Velocities systematically increased for the different stances (two-leg, one-leg, AP sway, ML sway, respectively). The effect of foam was not significant (p > 0.05).

**Fig 5:**
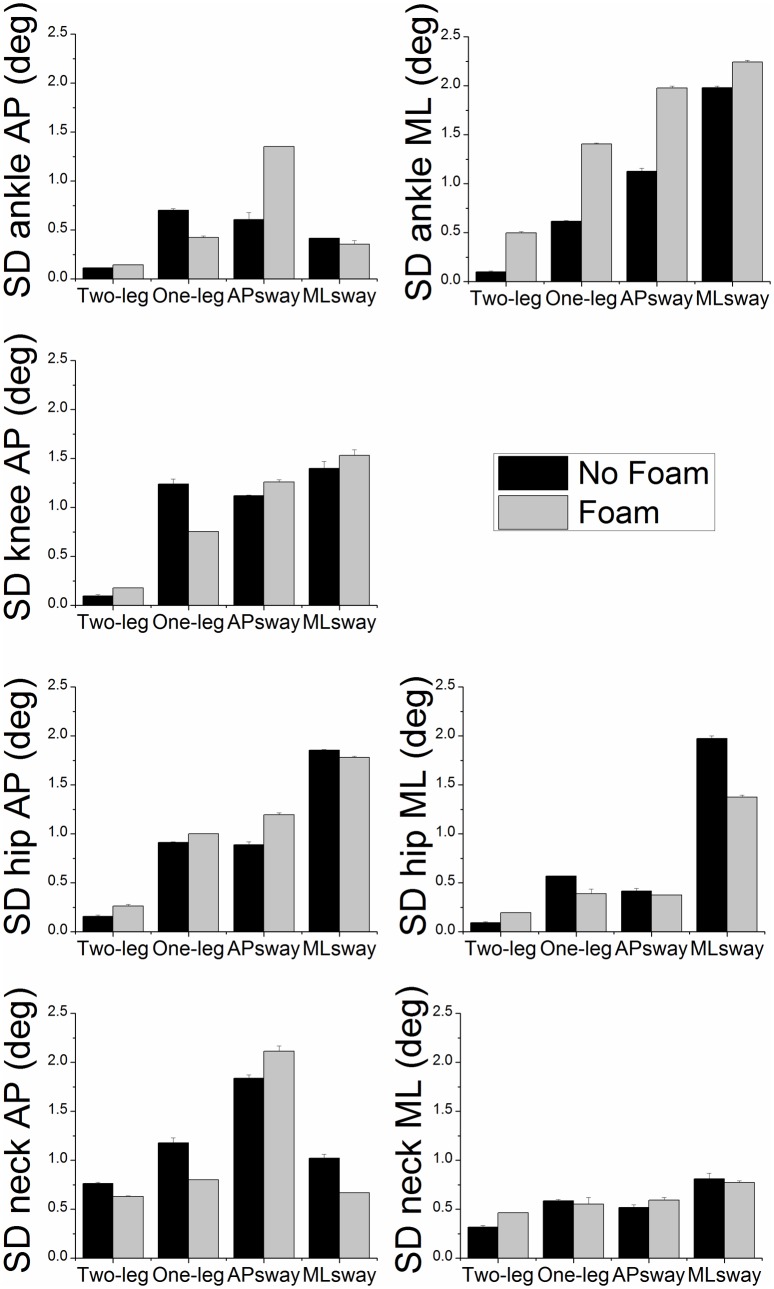
Circular SD (group means ± SE) of each joint motion (ankleAP, kneeAP, hipAP, neckAP, ankleML, hipML, neckML) as a function of postural stance and surface of support condition.

Fig 5 shows the circular SD of the joints that were included in the multi-joint-models. There were significant main effects of postural stance (ankleAP: F_3,33_ = 89.71, p < 0.01; kneeAP: F_3,33_ = 36.84, p < 0.01; hipAP: F_3,33_ = 41.24, p < 0.01; neckAP: F_3,33_ = 24.43, p < 0.01; ankleML: F_3,33_ = 53.33, p < 0.01; hipML: F_3,33_ = 62.68, p < 0.01; neckML: F_3,33_ = 22.31, p < 0.01). The SD of each joint motion generally increased during one-leg stance and during the dynamic tasks (AP and ML sway). Further, there were main effects of foam (ankleAP: F_1,11_ = 10.12, p < 0.05 and ankleML: F_1,11_ = 132.67, p < 0.01). SD of joint motion increased when standing on a foam surface of support.

### CCA model selection

The model building approach based on Wilks’ lambda [37] sequentially added the next best variable to the CCA model. The results have shown that 100% of the times the process continued until the last remaining variable. This means that the full 7DOF-model produced the best CCA model fit compared to subsets of the full model. The order of variable inclusion varied across trials and subjects.

**Fig 6:**
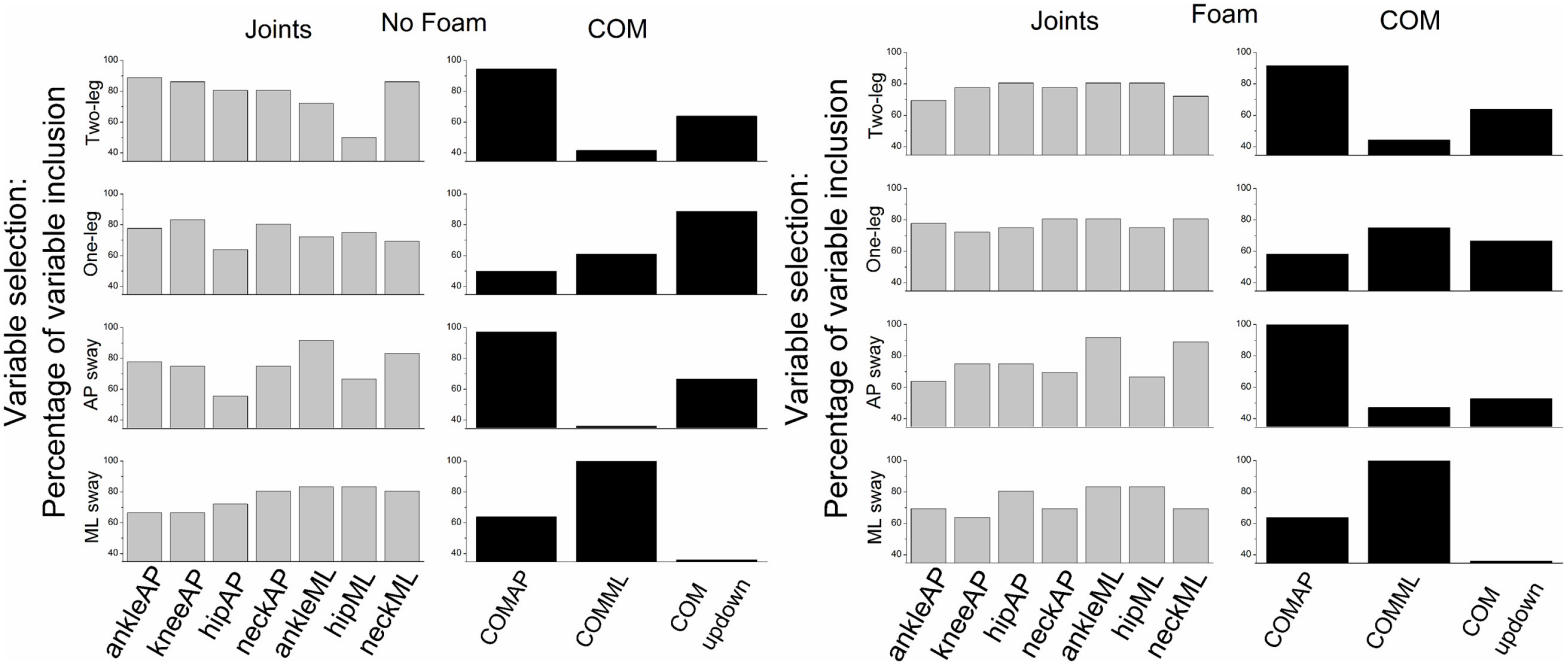
CCA variable selection: Selection of the best subset of the original variables based on the total CCA redundancy of the joint set. Percentage of variable inclusion in the best subset of the respective set across trials and participants is displayed for both joint and COM sets as a function of postural stance and surface of support condition.

The variable selection method based on the total CCA redundancy of *X* [38] produced 50–80% of the time best subsets X* of *X* that contained only 5 variables. The remainder of the times the best subsets X* contained 6 variables. The best subsets of *Y* were constrained to contain 2 variables. Fig 6 shows the percentages of variable inclusion in the best subsets X* and Y* as a function of postural stance and surface of support condition. The best subsets Y* showed in the main that COMAP and COMupdown sway were most important during two-leg and one-leg stances and voluntary AP sway, whereas COMML sway was most important during voluntary ML sway. For the other set, it appears that across participants and trials each variable was equally often included in the best subset X*. Note that the percentage of variable inclusion does not directly allow inference about variable importance once the variable was included in the best subset.

In the following, the 7DOF-model will be analyzed in detail as the model selection outcomes favored the 7DOF-model as the *best* model. In addition, only the first two canonical functions were analyzed. The rationale for this decision was that F-statistics have shown that the first two canonical functions were significant for every single trial and the 2DOF-model produced a maximum of 2 canonical functions.

### Total CCA redundancy index of 7DOF-model

**Fig 7:**
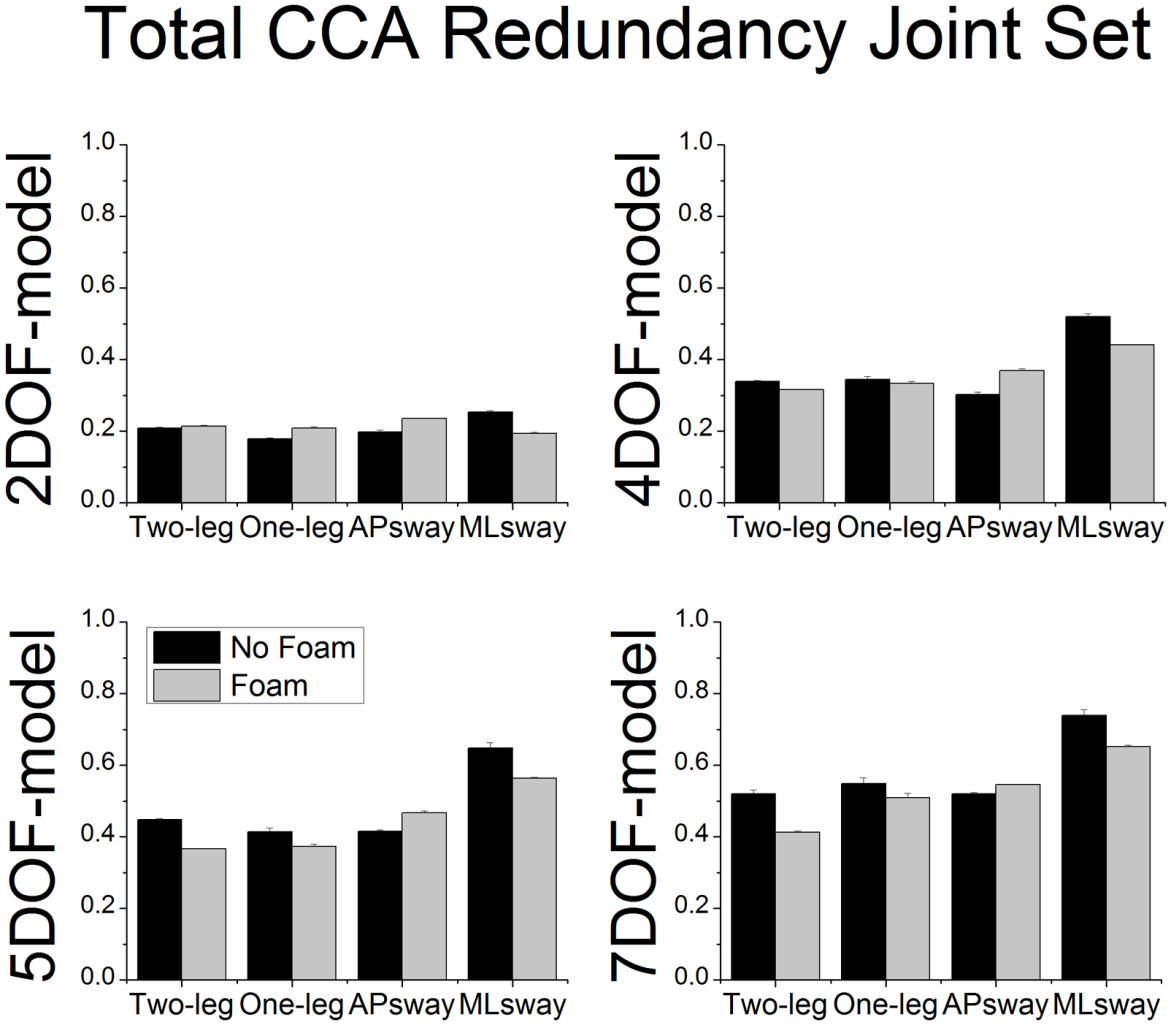
Total CCA redundancy (group means ± SE) of the joint set as a function of posture model, postural stance and surface of support condition. The total CCA redundancy of the theoretically motivated 2DOF, 4DOF and 5DOF-models were normalized to the 7DOF posture model.

**Fig 8:**
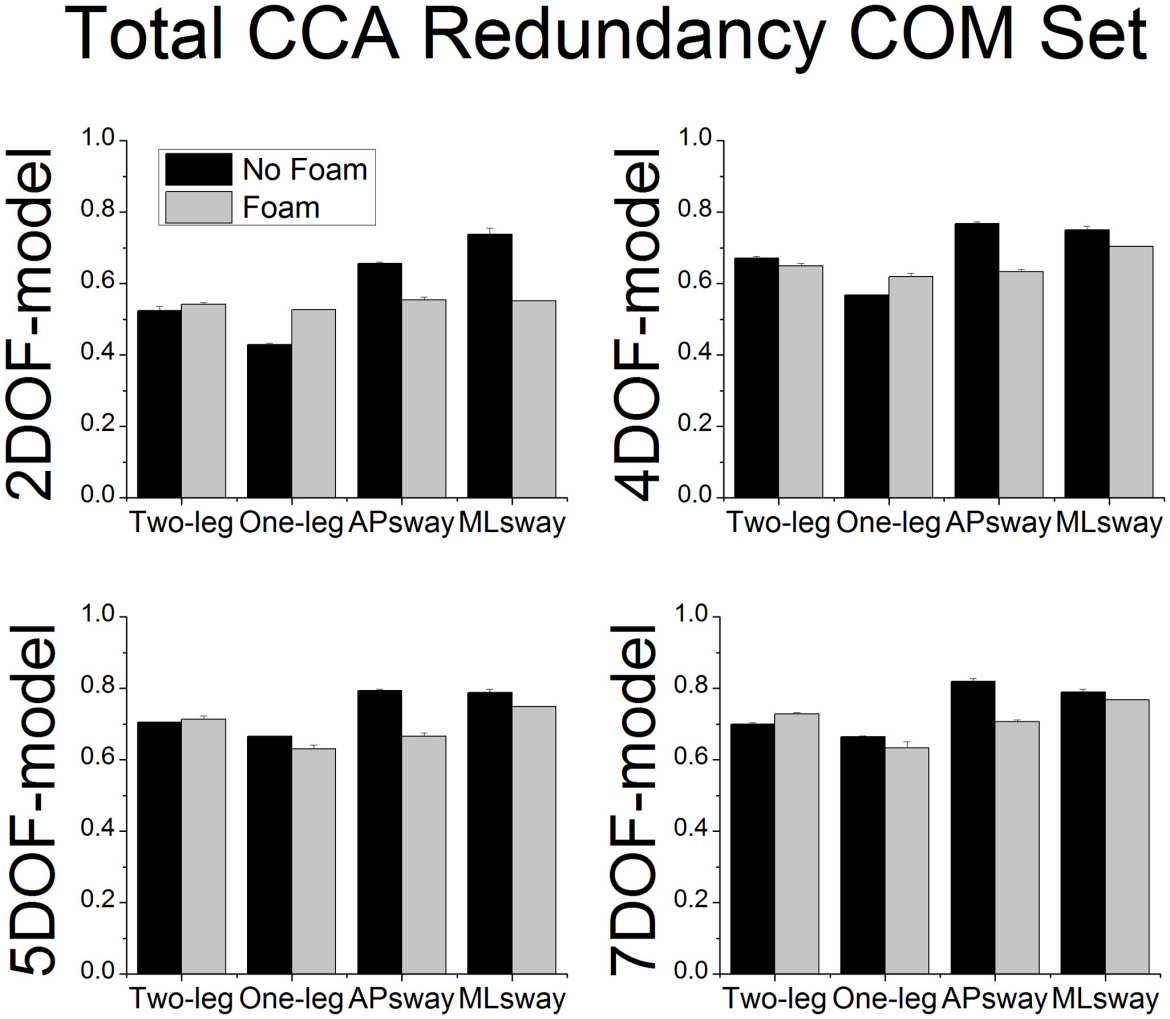
Total CCA redundancy (group means ± SE) of the COM set as a function of posture model, postural stance and surface of support condition.

There were significant main effects of postural stance (F_3,33_ = 7.97, p < 0.01) and foam (F_1,11_ = 14.78, p < 0.01) for the total CCA redundancy index of the joint set (Fig 7). The redundancy was lower under the foam conditions compared to no foam. Further, the redundancy was also lower for two-leg and one-leg stance compared to ML sway. For the total CCA redundancy index of the COM set (Fig 8) there were also significant main effects of postural stance (F_3,33_ = 3.74, p < 0.05) and foam (F_1,11_ = 12.01, p < 0.01). When standing on foam the redundancy decreased. The redundancy also decreased during one-leg stance compared to AP sway. In addition, the CCA redundancy indices of the theoretically motivated 2DOF, 4DOF and 5DOF models are also displayed in Figs 7 and 8. However, no statistical analysis was performed on these models as the model selection outcomes favored the 7DOF-model as the best model.

### CCA cross-loadings of 7DOF-model

**Fig 9:**
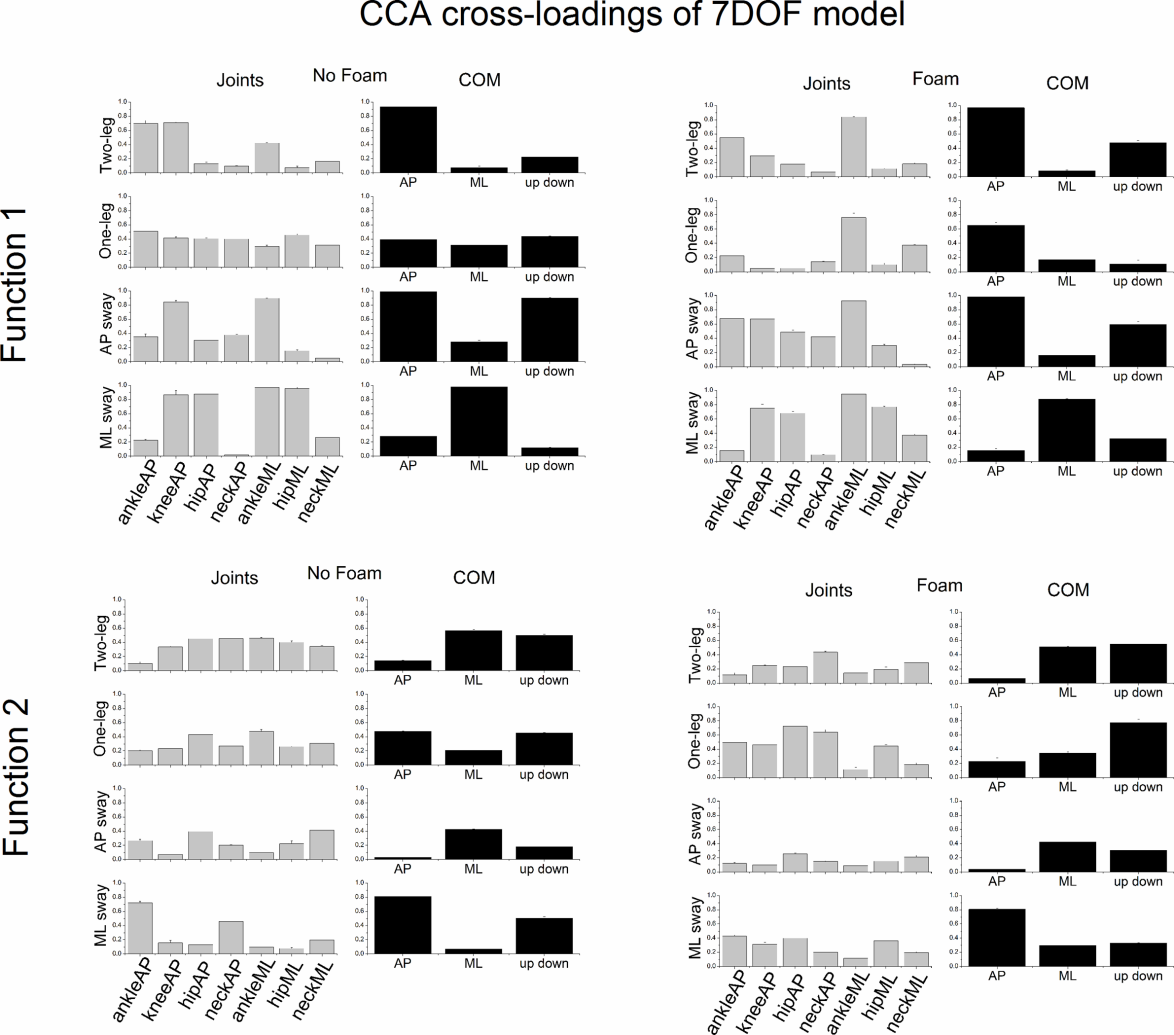
CCA cross-loadings (group means ± SE) of all variables of both joint and COM sets of the 7DOF model as a function of postural stance and surface of support condition. The cross-loadings of the first canonical function are displayed in the upper panels and the cross-loadings of the second canonical function in the lower panels.

Fig 9 shows the CCA cross-loadings of the 7DOF-model (both joint and COM sets) of the first two canonical functions as a function of stance and foam. Overall, high cross-loadings of function 1 decreased in function 2 and lower loadings increased. The following joint angular motions showed strikingly high loadings in function 1: ankleAP, kneeAP and ankleML during two-leg stance; ankleML and neckML during one-leg stance on a foam surface; kneeAP and ankleML during voluntary AP sway; ankleAP, kneeAP and ankleML during voluntary AP sway on foam; and finally kneeAP, hipAP, ankleML and hipML during voluntary ML sway. The following COM sway directions showed high cross-loadings in function 1: COMAP during two-leg stance and one-leg stance on foam, COMAP and COMupdown during AP sway and COMML during ML sway. One-leg stance on a rigid surface showed more uniform cross-loadings of all three variables. Finally, AP sway produced the lowest cross-loadings in function 2.

**Fig 10:**
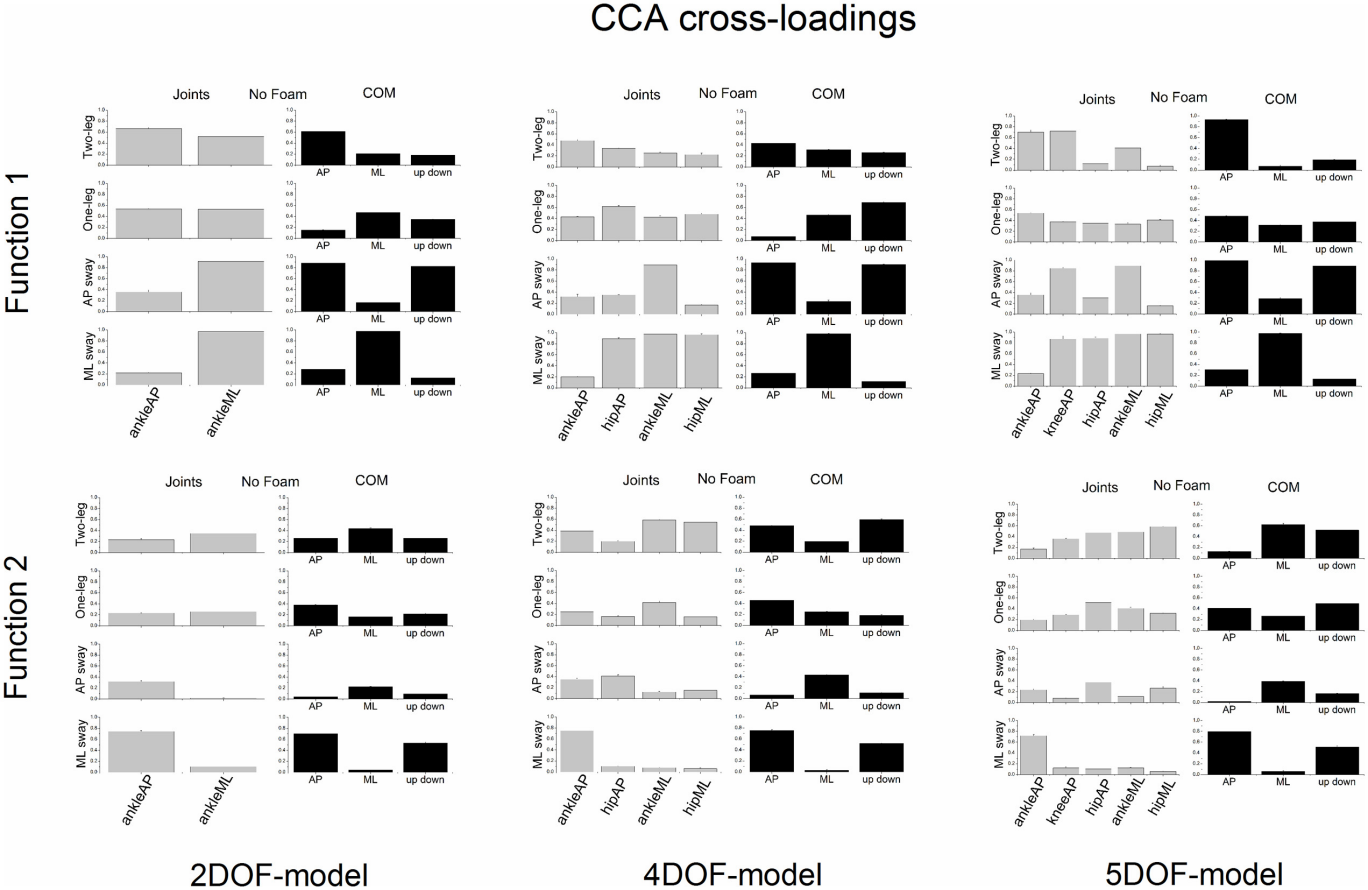
CCA cross-loadings (group means ± SE) of all variables of both joint and COM sets of the 2DOF, 4DOF and 5DOF models as a function of postural stance when standing on a rigid surface of support (No Foam). The cross-loadings of the first canonical function are displayed in the upper panels and the cross-loadings of the second canonical function in the lower panels.

**Fig 11:**
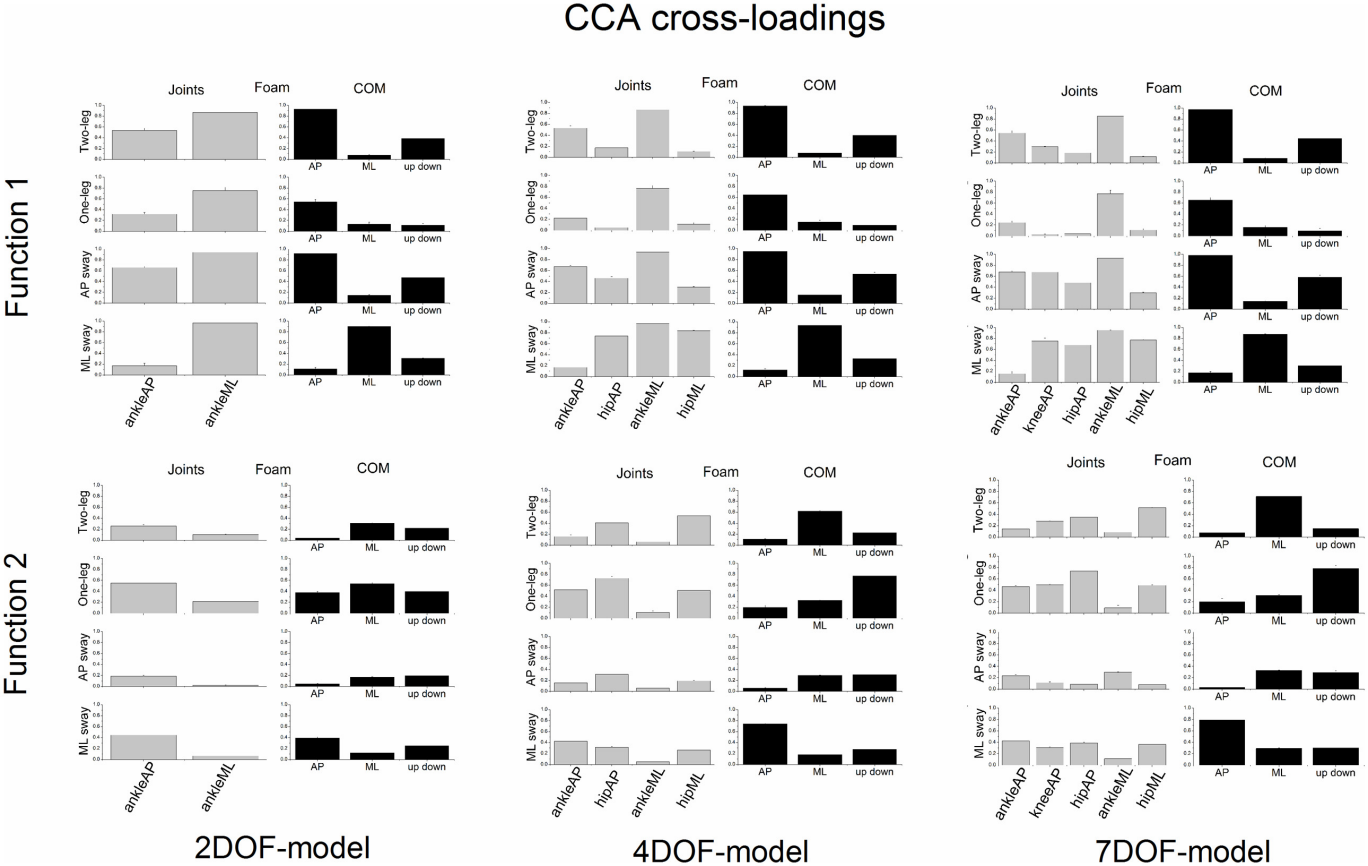
CCA cross-loadings (group means ± SE) of all variables of both joint and COM sets of the 2DOF, 4DOF and 5DOF models as a function of postural stance when standing on a foam surface of support (Foam). The cross-loadings of the first canonical function are displayed in the upper panels and the cross-loadings of the second canonical function in the lower panels.

Figs 10 and 11 show the CCA cross-loadings of the 2DOF, 4DOF and 5DOF models. In general, the results indicated that a high cross-loading of a particular variable was consistently high across models.

## Discussion

This study investigated the organization of the joint DOFs postural control system in different upright stances. Recent work has established that there are multivariate joint inputs of posture control [20–22, 25, 28] in contrast to the long-standing view of a single link inverted pendulum model [4], but the nature of the multivariate control and its relation to postural sway is still an open challenge. Here we used linear multivariate canonical correlation analysis [34–36] to *directly* determine the shared variance in the joint motions and the 3D motion of COM as a function of established models of postural control that varied in their joint DOF. This afforded a direct examination of the control of COM motion as a function of different multivariate inputs that varied in assumptions about joint DOF control: namely, an inverted pendulum ankle model (2 DOF), ankle-hip model (4 DOF), ankle-knee-hip model (5 DOF), and ankle-knee-hip-neck model (7 DOF).

Postural motion (COPnet and COM velocities) and joint motion variability systematically increased across progressively less stable stance conditions (bipedal quiet stance, one-leg stance, voluntary AP and ML sway). Variability of joint motions also either increased or decreased respectively when standing on a foam surface compared to a rigid surface of support. These findings are consistent with the proposition that the postural control system becomes more unstable with increasingly challenging constraints to upright stance and that this greater instability is accompanied by an enhanced level of activity at each individual joint, namely, ankle, knee, hip and neck [22]. More generally, these results provide further evidence that the upright human body moves as a multi-link system in order to maintain balance [15, 19, 20, 22, 28].

However, irrespective of the amount of variability (dispersion) the level of synchronization between COM and joint motions has the potential to reveal the functional contribution of the respective joint angular displacements in controlling COM position [4, 14]. Here, the total CCA redundancy index quantifies the shared variance of the motion of the joint components with that of 3D COM [34–36], that is, it estimates how much COM motion depends on the joint motions when both multivariate data sets are considered collectively. It follows that similar to R^2^ in multiple regression a higher CCA redundancy reflects increased predictability.

The total CCA redundancy of the joint set was higher for voluntary ML sway compared to one or two-leg stances and the total CCA redundancy of the COM set was higher for voluntary AP sway compared to one-leg stance. This shows that for the dynamic postural trials the relationship between joint and COM sets was greater in terms of the proportion of total variance that was explained by the first two canonical functions. We conclude that the dimensionality may be reduced in the dynamic conditions and thus the postural control coordination solution simplified [42]. In our case this means that the controlled DOF are lower than the dimensions of the data sets. In a similar way the CCA redundancy index of both sets was also higher when standing on a rigid ground support compared to a foam surface. The finding that the first two canonical functions captured less shared variance when standing on foam reflects an increase in the dimension of the postural control strategies [10, 25].

Based on the normalized total CCA redundancy index of the joint sets it was found that the full model (7DOF) accounted for a greater shared variance between the two sets of variables than the theoretically motivated subsets (2DOF, 4DOF and 5DOF models). Total shared variances of the first two canonical functions of the 7DOF model ranged from 40–70%, which is considered to be high in the context of CCA [34]. This observation was supported by the finding that the subset out of all possible subsets of the full model that best reproduced the shared variance of the full model contained at least 5 variables. We conclude that models with fewer DOFs (e.g., an inverted pendulum-like model) are not sufficient to capture the critical shared variance of the original full DOF model. However, the fact that 50–80% of the time the best subsets contained one variable less than the maximum number of possible variables may reflect a reduction of the controlled DOF. In addition, the findings showed individual patterns across trials and subjects, which highlights that no joint angular motion can be a priori excluded from the model. Furthermore, similar to regression model building we found that the full 7DOF model produced the best canonical model fit compared to subsets. It appears that most of the joint angular motions directly contribute to the control of COM.

To gain deeper insight into the specific role of each joint motion in stabilizing COM position against gravity we analyzed the cross-loadings of all joints. Higher loadings imply that these joints play a major role as changes in the respective joint angle are directly linked to deviations of the COM position. In addition, the cross-loadings of the 3D COM showed the principal directions of postural sway. Generally we found that the contribution of each joint and the dominant COM sway direction varied across postural stance and surface of support conditions, revealing adaptive postural strategies [20, 22, 25]. The first canonical function was thereby considered to reflect the primary control mechanisms.

During bipedal quiet stance on a rigid surface we observed an ankle (both AP and ML directions)—knee strategy that primarily controlled COM AP sway. This outcome is consistent with previous work that showed during quiet two-legged stance that the ankle joint motion is most representative of COM sway in the sagittal plane [3–9, 25]. On the other hand, the finding of a substantial role of the knee over the hip joint [4, 17–19] challenges the proposition of ankle-hip synergy as dominating coupling relationship at the joint level [3, 5, 15, 16, 30].

For one-leg stance we found a strong multi-DOF strategy [25], that is, all joints co-varied with the COM position and all three directions of COM were equivalently important. Further, the first canonical function of the more dynamic trials (voluntary AP and ML sway) also revealed a postural strategy that involved contributions of variance from the ankle, knee, hip and neck joints. During AP sway kneeAP and ankleML correlated the most with COM AP and COM up down motion. Except for hipML and neckML the loadings of the remaining joints were also high. These results show that the task that most resembles the traditional single inverted pendulum model in the sagittal plane [2], did not produce an inverted pendulum-like ankleAP strategy but rather a multi-DOF postural control strategy with primary control in the AP direction given the task instruction. During ML sway we also found that all joints, except for neckAP controlled COM ML sway. Similarly to AP sway this set of outcomes reflects the organization of a multi-link postural system.

Moreover, it is noteworthy to highlight that it is in the more challenging and dynamic postures that the multi-DOF are involved in more active roles [10, 25, 26, 28, 43]. When standing on a foam surface, which generally has been shown to increase the overall postural sway [16, 29], the functional joint DOF for one-leg stance were reduced. Whereas on a rigid surface all joints equally contributed to control the 3D COM position, on foam solely neckML and the ankle joint highly correlated with COM AP sway. It appears that the postural control strategy of one leg stance is driven by the mechanical properties of the foam that produces enhanced ankle inversion—eversion instability. On the contrary, during voluntary AP sway on a foam surface the multiple DOF are more actively exploited.

The second canonical function captures the shared variance between the two sets under the constraint to be uncorrelated to the first function. In general, we observed a switch in loadings, that is, the loadings that were low in the first canonical function became higher in the second canonical function. Considering the first two canonical functions together, we conclude that each joint motion has an active role in controlling the different components of the 3D COM motion. Finally, CCA can be sensitive to changes in the data sets. However, when comparing posture models (2DOF, 4DOF, 5DOF and 7DOF) of this study, patterns of joint and COM cross-loadings were systematic. This outcome reflects a high degree of stability of the CCA analysis. Nevertheless, as CCA is a linear multivariate statistical method, non-linear relations among variables can only be captured to a first degree of approximation.

The concept of synergies and dimensionality reduction of the control problem have been discussed in the literature within the framework of the Uncontrolled Manifold (UCM) approach [11, 20–23, 44] and principal component analysis (PCA) [10, 25]. The UCM approach as described in Scholz and Schöner [23] is based on an a priori geometric model (but see de Freitas and Scholz [45]). In stable conditions that model can be applied to the variability across time of a multivariate time series obtained in a single replication; in dynamic conditions it is applied to the variance across replications at selected time points. The Jacobian at a chosen reference point is taken (local linearization) and, given that there is a difference in the dimension of the time series and the controlled DOF, the UCM-based decomposition is carried out. By varying the a priori geometric model (which DOF are presumed to be controlled) and testing for differences in the variances along the UCM versus the orthogonal space, the actual controlled DOF can be detected.

The described UCM approach holds similarities to a model-based PCA. It is based on linearization of the model and focuses on differences in explained variance. The details of the computations involved in the UCM approach compared to PCA are, however, quite different. PCA is a model-free linear transformation and simply maximizes the explained variance of the first component, then maximizes the explained variance of the second component, etc. From the UCM perspective, the PCA components that explain the most variance would initially seem to span the UCM, not the orthogonal space. But that interpretation would not hold in general. One has to be careful in specifying what kinds of variation are inherent in the observations. And, this depends on the details of the experiment in which the time series data have been obtained and the way in which the observed data are preprocessed.

In sum, the UCM approach is comparable to PCA if the experimental conditions generate stable behavior. If the latter is the case then the relation of the UCM method to CCA is comparable to the relation of PCA to CCA. That is, UCM/PCA decomposes the observed variance in a single set of multivariate time series, whereas CCA decomposes two distinct sets of time series in such a way that maximum linear prediction between the two sets is obtained using the first set-dependent components.

The CCA redundancy index reveals that there is degeneracy in the postural solutions at the level of joint space that is dependent on the stance and the surface of support. This is an inverse relation in the sense that a higher CCA redundancy score indicates a stronger direct relation between the independent and dependent data sets and hence a lower level of degeneracy to the joint space configuration. The dynamic postural task clearly shows greater predictability than the quiet standing task in the relation of the joint space solution to indices of postural sway.

The central issue of this paper was to examine the structure of the multivariate postural control system through a canonical correlation analysis [34–36]. Established models of postural control [2, 4, 5, 16, 19, 20, 22] that differed in their joint DOF were examined based on the most important shared variance between joint angular displacements and total body 3D COM motion. The purpose was to determine the nature of the functional DOF of ankle, knee, hip and neck joint motions. Based on CCA model selection procedures, the amount of shared variance and the cross-loading patterns we revealed the direct contribution of the multi-DOF mechanisms [20] to postural control. Furthermore, we observed a reduction in dimensionality during the dynamic posture conditions (voluntary AP and ML sway) as opposed to quiet stance and when standing on a rigid surface compared to foam, suggesting simplified postural control coordination solutions.
